# Drug Repurposing of the Unithiol: Inhibition of Metallo-β-Lactamases for the Treatment of Carbapenem-Resistant Gram-Negative Bacterial Infections

**DOI:** 10.3390/ijms23031834

**Published:** 2022-02-06

**Authors:** Vitaly G. Grigorenko, Maria G. Khrenova, Irina P. Andreeva, Maya Yu. Rubtsova, Anastasia I. Lev, Tatiana S. Novikova, Elena V. Detusheva, Nadezhda K. Fursova, Ivan A. Dyatlov, Alexey M. Egorov

**Affiliations:** 1 Department of Chemistry, Lomonosov Moscow State University, 119991 Moscow, Russia; Imtek1@mail.ru (I.P.A.); mrubtsova@gmail.com (M.Y.R.); alex.m.egorov@gmail.com (A.M.E.); 2 Bach Institute of Biochemistry, Federal Research Centre “Fundamentals of Biotechnology” of the Russian Academy of Sciences, 119071 Moscow, Russia; 3 State Research Center for Applied Microbiology & Biotechnology, 142279 Obolensk, Russia; anastasia.lev@weizmann.ac.il (A.I.L.); pozitifka.15@yandex.ru (T.S.N.); klub@bk.ru (E.V.D.); n-fursova@yandex.ru (N.K.F.); dyatlov@obolensk.org (I.A.D.)

**Keywords:** metallo-β-lactamase, drug repurposing, antibiotic resistance, molecular modeling

## Abstract

The increasing antibiotic resistance is a clinical problem worldwide. Numerous Gram-negative bacteria have already become resistant to the most widely used class of antibacterial drugs, β-lactams. One of the main mechanisms is inactivation of β-lactam antibiotics by bacterial β-lactamases. Appearance and spread of these enzymes represent a continuous challenge for the clinical treatment of infections and for the design of new antibiotics and inhibitors. Drug repurposing is a prospective approach for finding new targets for drugs already approved for use. We describe here the inhibitory potency of known detoxifying antidote 2,3-dimercaptopropane-1-sulfonate (unithiol) against metallo-β-lactamases. Unithiol acts as a competitive inhibitor of meropenem hydrolysis by recombinant metallo-β-lactamase NDM-1 with the K_I_ of 16.7 µM. It is an order of magnitude lower than the K_I_ for l-captopril, the inhibitor of angiotensin-converting enzyme approved as a drug for the treatment of hypertension. Phenotypic methods demonstrate that the unithiol inhibits natural metallo-β-lactamases NDM-1 and VIM-2 produced by carbapenem-resistant *K. pneumoniae* and *P. aeruginosa* bacterial strains. The 3D full atom structures of unithiol complexes with NDM-1 and VIM-2 are obtained using QM/MM modeling. The thiol group is located between zinc cations of the active site occupying the same place as the catalytic hydroxide anion in the enzyme–substrate complex. The sulfate group forms both a coordination bond with a zinc cation and hydrogen bonds with the positively charged residue, lysine or arginine, responsible for proper orientation of antibiotics upon binding to the active site prior to hydrolysis. Thus, we demonstrate both experimentally and theoretically that the unithiol is a prospective competitive inhibitor of metallo-β-lactamases and it can be utilized in complex therapy together with the known β-lactam antibiotics.

## 1. Introduction

The microbial resistance to antibiotics represents an urgent problem of clinical medicine and pharmacology [1,2,3]. The development of antibiotic resistance in bacteria is a biological process that protects bacteria from the action of other microorganisms [4]. However, the active use of antibiotics in medicine and agriculture served as a major factor in the selection of resistant strains [5]. The high dissemination and spread rate of resistant bacteria is determined by the plasmid localization of the resistance genes [6,7]. The development of bacterial resistance to β-lactams, the most actively used class of antibiotics, is governed by synthesis of β-lactamase enzymes (EC 3.5.2.6) [8,9]. 

Currently, the β-lactamase superfamily comprises more than 2700 genetically and functionally different enzymes capable of hydrolyzing the β-lactam ring of the antibiotics [10,11,12]. Based on the structure of the active site, all β-lactamases are divided into serine-type and metal-dependent enzymes with one or two zinc ions in the active site [13,14]. Metallo-β-lactamases (MBLs) represents a great concern as they are characterized by high catalytic activity against almost all β-lactams except monobactams [15]^.^ MBLs are divided into three subclasses, B1, B2, and B3, depending on the homology of the primary structure, substrate profile, and the number of zinc ions in the active site [13]. The enzymes of the B1 and B3 subclasses are bi-zinc, and of the B2 subclass are mono-zinc [14]. The active site composition and surrounding demonstrate high variability for different subclasses that complicates the search for novel broad spectrum inhibitors [16,17].

The most clinically relevant MBLs belong to the subclass B1, and their widest geographical spread is attributed to the NDM type enzymes [18]. Their increasing prevalence is complicated by association of their genes with other resistance markers resulting in conferring the resistance to all known antibiotics [3]. Protein globule of B1 subclass enzymes has a αβ/βα sandwich fold, the active site contains two zinc ions bridged by a catalytic hydroxide ion, and two flexible loops are located at the active site entrance. Two zinc ions participate in the orientation of the carbonyl group of the β-lactam ring and carboxyl group of the substrate. The hydrolysis reaction is initiated by the nucleophilic attack of the hydroxide ion on the carbonyl carbon atom of the β-lactam ring [19,20]. This process results in the antibiotic inactivation due to the cleavage of the C-N bond of the β-lactam ring [21,22,23].

A common approach to overcome the resistance caused by β-lactamases is combining the antibiotics with the inhibitors [24]. The search for MBL inhibitors is extremely intensive and involves various approaches targeting different structural elements of the enzymes. The most frequently developed approach concerns the search for compounds chelating zinc ions. Derivatives of boronic acids represent promising inhibitors of another type as they interact with the catalytic hydroxide anion forming enzyme–inhibitor complexes mimicking the tetrahedral intermediate of the antibiotic hydrolysis [20,25,26,27]. Additionally, inhibitors may target the loops surrounding the cavity of the active site [12,28]. Despite years of intensive research, and more than 500 potential inhibitors reported in literature, there is still no MBL inhibitor approved for clinical use [17,28]. The development of efficient inhibitors for these β-lactamases is limited by their structural diversity, which affects the active site and possible targets.

There are two major directions in the search for inhibitors: structure-based multi-step virtual screening using the libraries of millions of chemical compounds and targeted search for the inhibitors with selected properties. For MBLs, zinc ions are the most common target, and compounds containing one or several thiol groups demonstrate a high affinity for zinc cation and are actively studied as potential inhibitors [29,30,31]. One of the challenges of this approach is to ensure that the potential inhibitor is selective to the MBL and does not interact with other metal-dependent enzymes. Among various compounds that may form coordination bonds with zinc cations, special attention is paid to approved drugs. This concept is called drug repurposing and it is becoming popular in the pharmaceutical industry as the development and introduction of new drugs into clinical practice is time consuming and extremely expensive [32]. The fundamental interest in the search for new targets for “old” (reused) drugs lies in the deep understanding of the drug pathways and regulatory mechanisms. 

Eleven thiol-containing compounds approved as drugs were examined as inhibitors of MBLs NDM-1, VIM-1, and IMP-7 [33]. Maximal inhibition activity against all three tested MBLs was observed for L-captopril (ACE inhibitor approved for treatment of hypertension), thiorphan (metabolite of racecadotril, an enkephalinase inhibitor, approved for treatment of diarrhea), and dimercaprol (antidote approved for treatment of toxic metal poisoning). Dimercaprol, also known as British Anti-Lewisite (BAL), showed the lowest IC_50_ value of all drugs tested. BAL was also reported as an inhibitor of MBL NDM-1 [34]. This stimulated us to search MBL inhibitors among other heavy metal antidotes.

**Figure 1:**
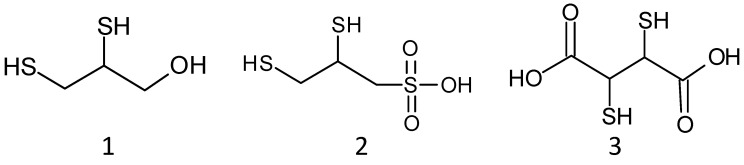
Structures of dimercaprol, BAL (1); unithiol, DMPS (2); DMSA (3).

Chelation therapy by antidotes is commonly used to treat metal toxicity [35]. Different chelating agents were examined toward their increased specificity for certain metal ions and the ability to penetrate through cell membranes. The compounds of interest should have enhanced affinity for metals compared with the normal body ligands, be able to pass the cell membranes, and be easily excreted from the body without any further interaction with the vital organs. Among them, the most actively studied are BAL and its analogues dimercaptopropanesulfonate (DMPS, unithiol) and dimercaptosuccinic acid (DMSA) (Figure 1). They all have two thiol groups that form a stable complex with metal toxicants. BAL was the first antidote against an arsenic nerve agent. However, its application revealed significant drawbacks associated with a narrow therapeutic window, limitations of intramuscular injection and a risk of allergic reactions. That is why BAL has been largely supplanted by unithiol and DMSA that have greater water solubility, lower toxicity, possibility of intravenous administration, and wider therapeutic activity [36,37]. Of interest is the fact that BAL, DMPS, and DMSA were able to inhibit MBL IMP-1 [38].

Herein, we report the results on the combined experimental and computational study of the thiol containing drugs repurposed for the MBLs inhibition. We tested in vitro a set of 10 compounds as potential inhibitors of the MBL NDM-1. The most prospective compound, unithiol, was further examined against multi-drug resistant Gram-negative bacteria producing MBLs NDM-1 and VIM-2 by the disk diffusion method. The mechanism of the unithiol binding was determined by enzymatic experiments and molecular dynamic simulations with the combined quantum mechanics/molecular mechanics potentials. 

## 2. Results 

### 2.1. Unithiol as an Inhibitor of Metallo-β-Lactamase NDM-1

We followed the strategy of drug repurposing to find new inhibitors of MBLs. For this purpose, we studied a set of chemical compounds (acetylcysteine, pentetate, unithiol and several bisphosphonates, including pamidronic acid) as potential inhibitors of recombinant MBL NDM-1. All of them have been approved for clinical use and have intrinsic chelating ability and, thus, may act as MBL inhibitors. Their effect in a concentration of 100 µM on the hydrolysis of the chromogenic substrate CENTA by recombinant NDM-1 is shown in Tables S1 and S2. Among them, unithiol possesses the most potent inhibitory ability. 

**Figure 2:**
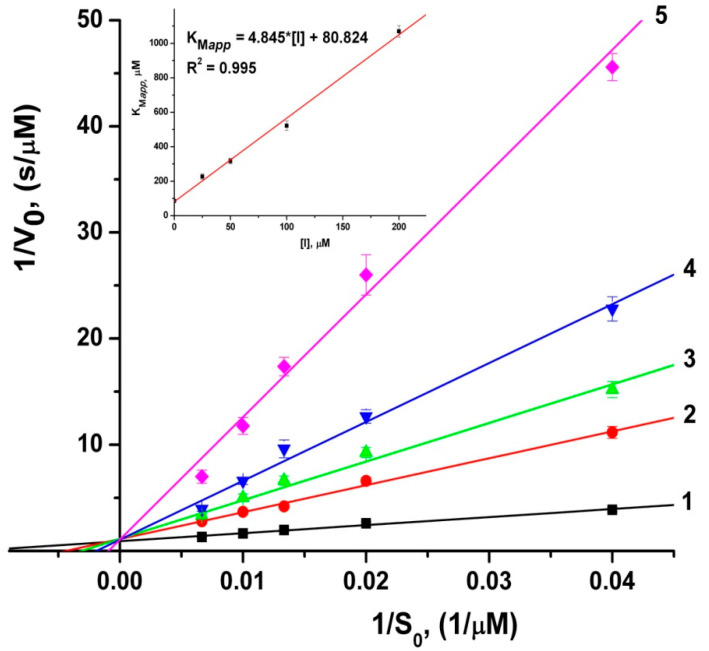
Double reciprocal 1/V_0_ versus 1/S_0_ Lineweaver–Burk plots for the meropenem hydrolysis by metallo-β-lactamase NDM-1 at different concentrations of unithiol: 1–0; 2–25; 3–50; 4–100; 5–200 μM. Initial concentrations of meropenem are 25, 50, 75, 100, and 150 μM. The insert shows the dependence of apparent Michaelis constant (K_Mapp_) on the concentration of unithiol, [I].

Parameters of the meropenem hydrolysis by recombinant MBL NDM-1 in the presence of unithiol were determined by enzymatic kinetics methods. Figure 2 represents the dependencies of the initial rates of meropenem hydrolysis at different concentrations of unithiol in the Lineweaver–Burk coordinates. It demonstrates the competitive mechanism of inhibition of recombinant MBL NDM-1 by unithiol. The inhibition constant is K_I_ = 16.7 ± 1.2 µM, that is similar to the IC_50_ value of 7.9 µM for D-captopril [34] and is one order of magnitude lower than that for l-captopril (202.0 µM) [39]. 

**Figure 3:**
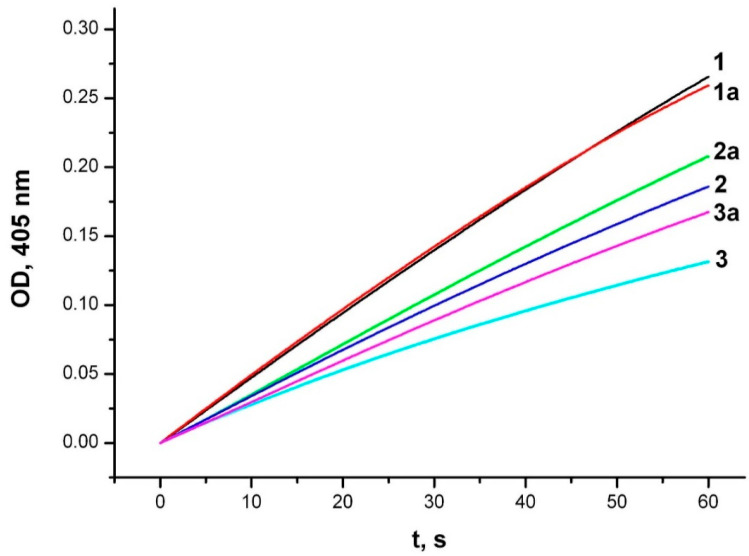
Inhibition of CENTA hydrolysis by metallo-β-lactamase NDM-1 in the presence of unithiol and zinc ions. Experimental conditions: initial concentrations of the NDM-1 (20 nM), CENTA (50 μM), 1—no unithiol and zinc ions; 1a—zinc ions (50 μM); 2—unithiol (20 μM); 2a—unithiol (20 μM) and zinc ions (50 μM); 3—unithiol (50 μM); 3a—unithiol (50 μM) and zinc ions (50 μM). The hydrolysis product accumulation is monitored in the 50 mM sodium phosphate buffer, pH 7.0 at 25 °C by continuous recording of the absorbance at 405 nm.

**Figure 4:**
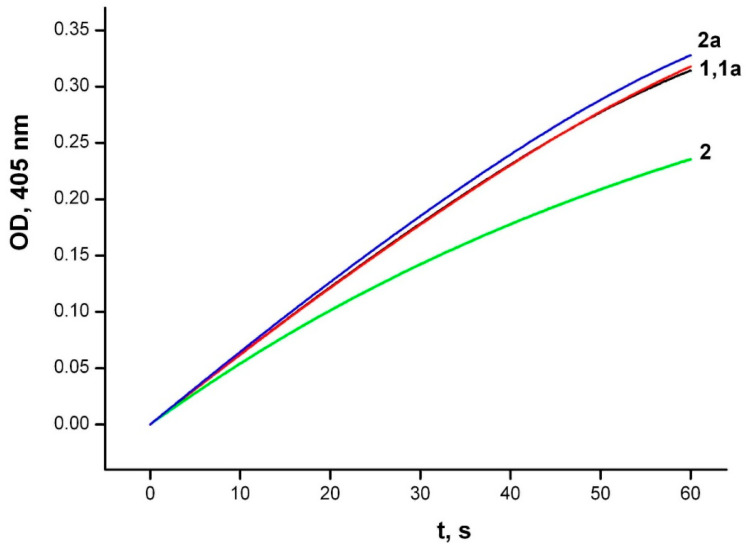
Inhibition of CENTA hydrolysis by metallo-β-lactamase NDM-1 in the presence of EDTA and zinc ions. Experimental conditions: initial concentrations of the NDM-1 (20 nM), CENTA (50 μM): 1—no EDTA and zinc ions; 1a—zinc ions (50 μM); 2—EDTA (20 μM); 2a—EDTA (20 μM) and zinc ions (50 μM). The hydrolysis product accumulation is monitored in the 50 mM sodium phosphate buffer, pH 7.0 at 25 °C by continuous recording of the absorbance at 405 nm.

The inhibitory effect of unithiol against MBL NDM-1 can be due to both its ability to form coordination bonds with zinc ions in the enzyme active site, and to interact with the amino acid residues of the active site. To identify the mechanism of inhibition we performed additional experiments. First, we compared the inhibition potency of the unithiol in the presence and absence of zinc ions in solution. Excessive concentration of zinc ions slightly reduced the inhibition by unithiol (Figure 3). Thus, the binding constant of unithiol to the NDM-1 enzyme is significantly higher than the binding constant to zinc cations in solution. Then, we compared the effects of unithiol and EDTA on the MBL under the same conditions (Figure 4). Adding EDTA to the reaction media decreases the hydrolysis rate similarly to the unithiol. However, the inhibition effect disappears when an excess of zinc ions is added to the solution. This means that EDTA does not have any specific interactions with the enzyme active site and acts simply as a zinc chelate. Contrary, the unithiol preferably binds to the active site of NDM-1, presumably forming specific contacts with the protein globule. To further investigate the molecular mechanism of the unithiol binding, we performed molecular simulations that are discussed in the following sections.

### 2.2. Effect of Unithiol and EDTA on the Metallo-β-Lactamases Produced by Bacterial Strains Resistant to Carbapenems

Further investigation of the ability of unithiol to inhibit natural MBLs was carried out using clinical strains of Gram-negative bacteria resistant to carbapenems. Phenotypic and genotypic data for the strains selected for this study are described in the Experimental Section. Among them, two strains of *K. pneumoniae* (409 and 410) produce MBL NDM-1 and two strains of *P. aeruginosa* (B-2099/18 and B-730P/17) produce MBL VIM-2. 

Kinetic analysis was performed using periplasmic fractions of *P. aeruginosa*, containing active mature MBL VIM-2. The choice of this enzyme was due to the availability of clinical strains producing only this single MBL in contrast to clinical producers of NDM-1, co-expressing a mixture of different β-lactamases. A noticeable dose-dependent inhibitory effect of unithiol (Figure S1) was observed for the two strains studied (*P. aeruginosa* strains B-730P/17 and B-2099/18).

**Figure 5:**
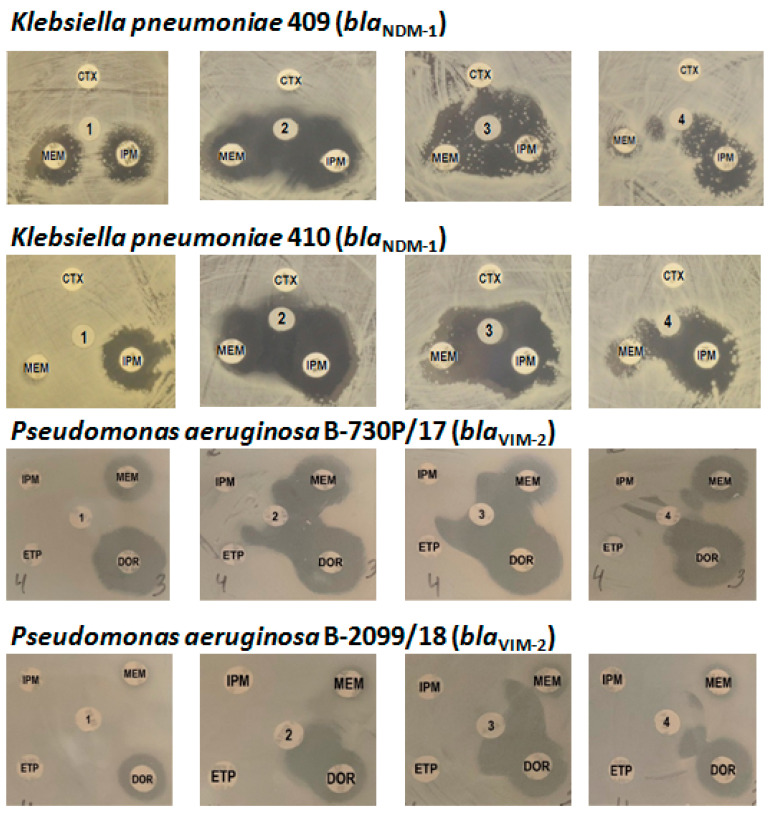
Phenotypic detection of metallo-β-lactamases NDM-1 and VIM-2 inhibition by unithiol and EDTA by the disk diffusion method: 1, saline; 2, EDTA 730 µg; 3, unithiol 3770 µg; 4, unithiol 470 µg; CTX, cefotaxime; MEM, meropenem; IPM, imipenem; DOR, doripenem; ETP, ertapenem.

Then the inhibition activity of unithiol was studied directly on bacterial cultures by the disk diffusion method, and EDTA, a reference MBL inhibitor, was used as a positive inhibition control (Figure 5). In the absence of inhibitors, bacterial growth was observed in the presence of control antibiotic cefotaxime, the III generation cephalosporin. In the presence of carbapenems, only weak suppression of cell growth was observed. The inhibitory effect, i.e., the increase in the zone in which the cells did not grow, was observed both in the presence of EDTA and unithiol. Moreover, a dose-dependent effect was observed: the 2-fold increase in the inhibition zone around the discs containing unithiol (3770 and 470 µg/disk). The effect was comparable to the inhibitory effect of EDTA (730 µg/disk). It was revealed that unithiol protected imipenem and doripenem from the hydrolysis by MBLs NDM-1 and VIM-2 more effectively than meropenem.

The results obtained by the disk diffusion method were confirmed by studying the effect of unithiol on the minimum inhibitory concentrations (MICs) of antibiotics for two bacterial strains resistant to carbapenems with the broth microdilution method. To determine the concentration range of unithiol, its own MIC was determined, which was 6.3 mg/mL (Table 1). Since bacterial growth is suppressed by unithiol at concentrations exceeding the MIC value, the concentrations below the MIC were further investigated. It was shown that the MICs of meropenem and imipenem for *K. pneumoniae* strain 410 (*bla*_NDM-1_) decreased by 4–16 times in the presence of 1.5 and 3.1 mg/mL of unithiol compared to the initial values (Table 1), which is considered significant for this method. Similarly, MICs of doripenem and ertapenem for *P. aeruginosa* strain 730P/17 (*bla*_VIM-2_) decreased by four times in the presence of the same concentrations of unithiol. It can be proposed that the decrease in the MICs of carbapenems is due to the inhibition of MBLs by unithiol.

Thus, unithiol showed synergistic inhibitory activity against MBLs NDM-1 and VIM-2 produced by clinical strains in phenotypic tests using different carbapenems. Since phenotypic tests showed the inhibitory effect of unithiol with respect to two different carbapenemases of molecular class B, molecular modeling and analysis of the complexes of unithiol with MBLs NDM-1 and VIM-2 were further carried out. 

### 2.3. Comparison of the Metallo-β-Lactamases NDM-1 Complexes with the Meropenem and Unithiol

**Figure 6:**
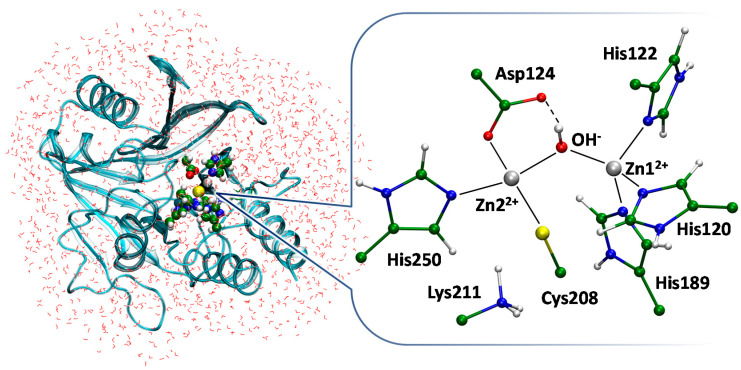
The overall structure of metallo-β-lactamase NDM-1 obtained in the QM/MM calculations. The rectangular water box is truncated to a smaller shell for clarity. Water molecules are shown in lines, a secondary structure of NDM-1 is shown in cartoon representation, and residues of the active site are shown by Van der Waals spheres. The inset demonstrates the active site. Histidine residues form coordination bonds with zinc cations both in apo form and in complexes with substrates or inhibitors. Here and on the next figures coordination and hydrogen bonds are shown by solid and dashed lines; the color code is the following: carbon in green, hydrogen in white, sulfur in yellow, oxygen in red, nitrogen in blue, zinc in silver.

**Figure 7:**
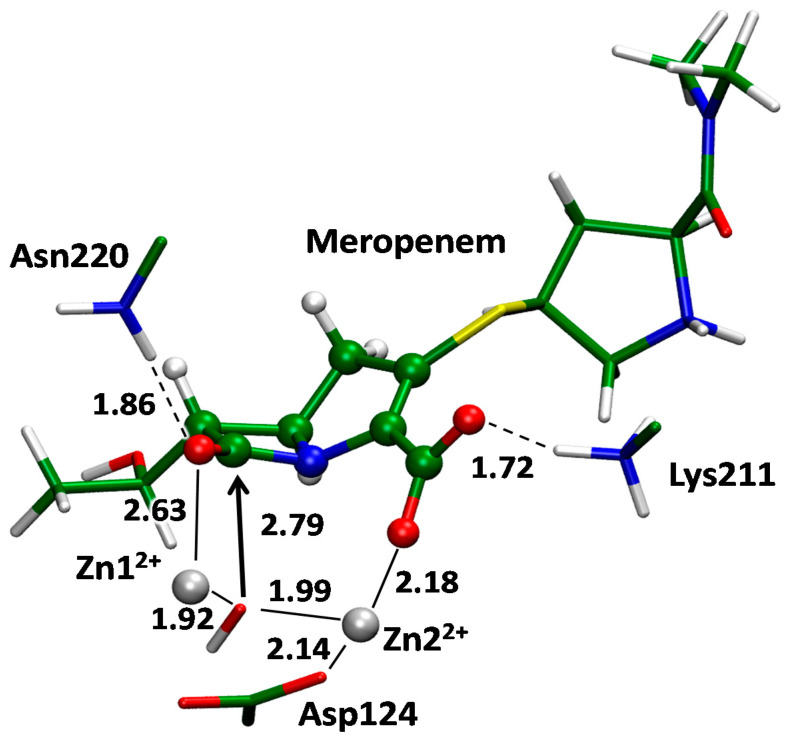
The enzyme–substrate complex of metallo-β-lactamase NDM-1 with meropenem. Here and on the next figures distances are shown in Å.

We obtained two complexes of MBL NDM-1 with its substrate meropenem and inhibitor unithiol in the active cite. These QM/MM models were obtained starting from the crystal structure of the MBL NDM-1 with the hydrolyzed imipenem in the active site (PDB ID: 5YPI) [22]. An active site of the MBL NDM-1 is shown on Figure 6. It shares common features of MBLs of subclass B1 and carries two zinc cations that polarize and orient catalytic OH^−^ species. Both zinc cations have tetrahedral coordination spheres; it is formed by the side chains of His122, His120, His189 and a catalytic OH^−^ for Zn1^2+^ and by side chains of Asp124, Cys208, His250 and a catalytic OH^−^ for Zn2^2+^. Coordination number of 4 is typical for Zn^2+^ in proteins [40]. Positively charged amino group of Lys211 is conservative for MBLs of subclass B1 and it coordinates carboxylate of all β-lactam antibiotics [22]. When the β-lactam is bound to the active site of the MBL, the coordination number of the Zn1^2+^ (for NDM-1) is increased to 5. The additional coordination bond is formed with the carbonyl oxygen atom of the four-membered ring of an antibiotic to activate the carbonyl group for the nucleophilic attack [20]. A catalytic OH^−^ forms a hydrogen bond with the side chain of Asp124 that is involved in the proton transfer during the hydrolysis (Figure 7). Following is the comparison of the EI and ES complexes between the NDM-1 and unithiol or meropenem, respectively. 

**Figure 8:**
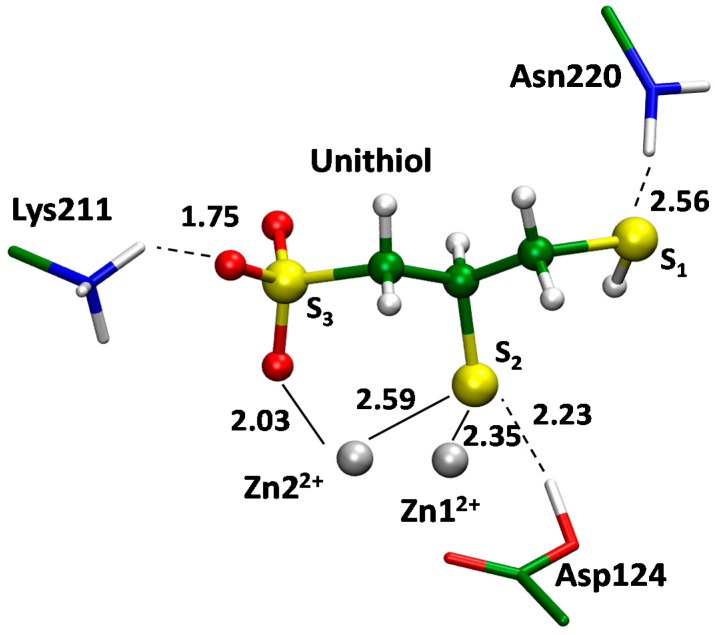
The enzyme–inhibitor complex of metallo-β-lactamase NDM-1 with unithiol.

The QM/MM modeling was utilized to obtain the full-atom 3D structure of the enzyme–inhibitor (EI) complex of the unithiol in the active site of MBL NDM-1 (Figure 8). We started with the analysis of the available X-ray data on the NDM-1 complexes with inhibitors. Compounds that carry thiol groups are bound so that the sulfur atom is located between zinc cations and coordinates both of them. It is observed for both MBLs, NDM-1 and VIM-2, that belong to the same B1 subclass of the enzymes [41,42]. Recently published crystal structures PDB ID: 5ZJ8 and 5ZJC reveal binding of two structurally similar compounds, (2S)-2,3-dithiolpropan-1-ol and (2S)-2-methyl-3-thiol-propan-1-ol, respectively. One of them has an additional thiol substituent and another one has a methyl group instead. Importantly, addition of the second thiol group does not change the binding mechanism as only one thiol group interacts with zinc cations. These experimental observations are in line with our model of the unithiol complex with the MBL NDM-1 (Figure 7). One of the thiol groups of a unithiol molecule is located between zinc cations and coordinates both of them; another thiol group does not have specific interactions with the NDM-1. The thiol group located between zinc cations simultaneously transfers its proton to the catalytic Asp124 upon binding. Negatively charged sulfate is oriented towards Lys211 and forms a hydrogen bond with its positively charged side chain. It also forms a coordination bond with a Zn2^2+^ cation. The S1 atom of unithiol forms a hydrogen bond with the side chain of Asn220. Thus, an inhibitor molecule is tightly bound to the active site by hydrogen and coordination bonds.

We compared interactions between the unithiol inhibitor and MBL NDM-1 in the EI complex (Figure 8) and the meropenem substrate in the active site of NDM-1 in the ES complex (Figure 7) using the same QM/MM methods. In the ES complex, a catalytic OH- species is located between zinc cations and its position is occupied by the –S- group of unithiol in the EI complex. The OH- forms a hydrogen bond with the Asp124 in ES whereas the proton is transferred simultaneously from the –SH group to the Asp124 along the hydrogen bond in the EI complex. In the EI complex, Lys211 interacts with the sulfate of unithiol and similarly it forms a hydrogen bond with the carboxylate of the meropenem in ES. The carboxylate of the Asp124 forms a coordination bond with a Zn2^2+^ cation in the ES complex. In the EI complex this coordination bond is broken and a novel coordination bond between a Zn2^2+^ cation and an oxygen atom of the sulfate group is formed. Thus, the unithiol molecule is a partner for different types of interactions in the active site of the NDM-1 being an efficient inhibitor. It should be mentioned that some interactions are stronger in the EI complex compared with the ES complex. The S- is the most preferable ligand for Zn^2+^; it serves as a ligand in the zinc finger motif that is known to be extremely stable [40]. It seems that the oxygen atom of the unithiol sulfate group is a more preferable partner for coordination bond compared with the neutral carboxylic group of Asp124 as the latter is substituted from the coordination shell upon formation of the NDM-1–unithiol complex.

### 2.4. The Metallo-β-Lactamase VIM-2–Unithiol Complex

**Figure 9:**
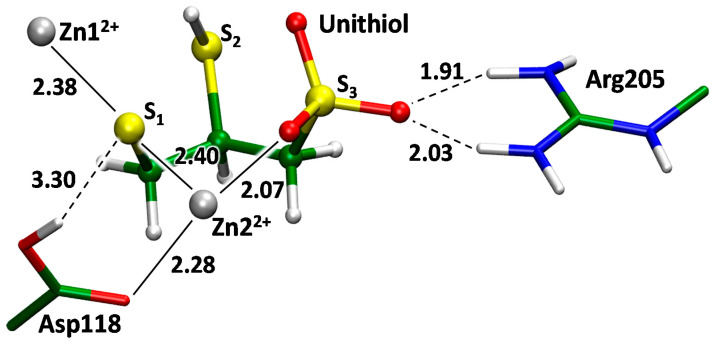
The enzyme–inhibitor complex of metallo-β-lactamase VIM-2 with unithiol.

The inhibitory effect of unithiol against MBL VIM-2 was demonstrated experimentally on periplasmic fractions isolated from *P. aeruginosa* strains B-730P/17 and B-2099/18 (Figure S1), therefore, we constructed the molecular model of the VIM-2–unithiol complex as well (Figure 9). The full atom molecular model with the VIM-2 was constructed by motifs of a crystal structure (PDB ID: 6J8R) [43]. A VIM-2 enzyme belongs to the same B1 subclass of MBLs as NDM-1 [44]. It has the same coordination shells for both Zn1^2+^ and Zn2^2+^ cations, but it lacks a lysine residue (Lys211 in NDM-1) that is responsible for orientation of the substrate due to the formation of hydrogen bonds with its carboxylate (Figure 7). This position is occupied by Tyr201 in MBL VIM-2. However, another positively charged residue, Arg205, is presented in this region. According to the QM/MM calculations it acts as a hydrogen bond partner for the sulfate group of the unithiol upon formation of the VIM-2–unithiol complex. The guanidinium group of Arg205 is located further from the active site in VIM-2 compared with the protonated amino group of Lys211 in the NDM-1. Therefore, the S1 atom of the thiol group of a unithiol molecule is located between the zinc cations (Figure 9). In MBL NDM-1 this location is occupied by the S2 atom of the unithiol thiol group that is closer to the unithiol sulfate group (Figure 7). 

## 3. Discussion and Conclusions

Antibacterial resistance of infectious pathogens is one of the most important public health problems worldwide. In recent years, this problem has become even more threatening due to the spread of pan-resistant bacteria producing various MBLs, capable of hydrolyzing most of the known β-lactam antibiotics. Numerous studies have been conducted to find novel antibacterial drugs including the compounds that are not inactivated by β-lactamases or can inhibit them. However, introducing new antibacterial drugs to the market is time-consuming and expensive. Moreover, there are still no metallo-β-lactamase inhibitors approved for clinical use. The so-called drug-repurposing is an attractive alternative that assumes the search for new targets of known drugs. 

In this study, it was shown that the unithiol, that is being used in clinical practice as an antidote against heavy metal poisoning, exhibits a new activity—inhibition of MBLs, in particular carbapenemases NDM-1 and VIM-2. A reversible competitive mechanism of inhibition by unithiol has been established for the hydrolysis of meropenem by recombinant enzyme NDM-1. The results obtained show a new target for drug repurposing of unithiol, similar to that found earlier for the approved drugs BAL and l-captopril. Moreover the inhibition activity of unithiol is comparable or higher by one order of magnitude compared to them [45,46]. 

Molecular modeling performed in this study further provides additional support and details of the competitive inhibition mechanism of unithiol. One of the sulfur atoms of the thiol groups locates between zinc cations and occupies the same space as the catalytic hydroxide anion in the enzyme–substrate complex does. Simultaneously, the proton from the thiol groups transfers to the catalytic aspartate that also stabilizes the complex. The sulfate group forms both a coordination bond with a zinc cation and a hydrogen bond with the positively charged residue, lysine or arginine. MBLs NDM-1 and VIM-2 have different active sites and different thiol groups are located between zinc cations. Therefore, the presence of two thiol groups in the unithiol is crucial for acting as an inhibitor in the differently shaped active sites. The catalytic OH^−^ in the complexes of unithiol and MBLs is substituted by the thiol group of an inhibitor, that is larger. Therefore, we checked how it changes the active site with respect to the distance between two zinc cations. According to the high-resolution X-ray data (~ 1 Å resolution), the Zn1^2+^…Zn2^2+^ distance in MBL NDM-1 in the absence of substrate or inhibitor is 3.5–3.6 Å [47]. In the QM/MM model of the NDM-1–unithiol complex, this distance is equal to 3.5 Å, thus the active site is not perturbed upon unithiol binding. The Zn1^2+^…Zn2^2+^ distance in the QM/MM VIM-2–unithiol is much larger and equals 4.5 Å, that is about 1 Å larger than in the unbound state according to the X-ray data [43]. 

Finally, we studied the inhibitory effect of the unithiol on Gram-negative bacteria involving multidrug-resistant strains that produce various MBLs including carbapenemases NDM-1 and VIM-2. Disk diffusion and broth microdilution methods demonstrate that unithiol inhibits native MBLs NDM-1 and VIM-2 produced by carbapenem-resistant *K. pneumoniae* and *P. aeruginosa* bacterial strains.

To conclude, we found that the unithiol inhibits carbapenemases both in vitro and in vivo. It can be utilized in complex therapy with the known β-lactam antibiotics. If inhibited by the unithiol, MBLs will not inactivate β-lactams and the latter can achieve their targets, penicillin binding proteins.

## 4. Materials and Methods

### 4.1. Materials

The reagents were purchased from Sigma-Aldrich (St. Louis, MO, United States), ICN Biomedicals (Costa Mesa, CA, United States), Difco (BD, New York, NY, United States), Gibco (Thermo Fisher Scientific, Waltham, MA, United States), and Khimmed (Moscow, Russia). All buffer solutions were prepared using deionized Milli-Q water (Millipore, Molsheim, France). The following β-lactamase substrates were used: meropenem, imipenem, doripenem, ertapenem, cefotaxime (Oxoid, Cambridge, UK). All solvents and chemicals were used as purchased without further purification.

### 4.2. Protein Isolation and Purification

Isolation and purification of recombinant MBL NDM-1 was carried out as described previously [48]. In brief, the periplasmic fraction of *E. coli* BL21(DE3) cells, transformed with pET-NDM vector, bearing gene coding for the NDM-1 enzyme, was liberated by an osmotic shock procedure and further purified by anion-exchange chromatography. The enzyme was stored at 4 °C.

### 4.3. Measuring the Kinetic Parameters and the Inhibition Constants of the Recombinant Metallo-β-Lactamase NDM-1

The activity of MBL NDM-1 was determined toward the chromogenic substrate CENTA and antibiotic meropenem using a UV-1602 spectrophotometer (Shimadzu, Japan) at 25 °C in 50 mM sodium-phosphate buffer solution (pH 7.0) supplemented with zinc ions (50 µM). The total volume of the enzyme assay was 1 mL. The concentration of the enzyme in the assay was 20 nM. The initial substrate concentrations ranged from 10 to 400 mM. The concentrations of unithiol in the assay were 25, 50, 100, and 200 μM. Product formation was detected at the wavelength of 405 nm (Δε_405_ = 6400 M^−1^ cm^−1^) for CENTA and 300 nm (Δε_300_ = −10940 M^−1^ cm^−1^) for meropenem. All measurements were repeated at least three times. The k_cat_ values were taken as the *V*/[E] ratio, where [E] is the enzyme concentration.

Apparent Michaelis constants (K_Mapp_) and V_max_ were determined using a weighted Lineweaver–Burk linearization. The weights were taken as V_0_^4^/σ^2^(V_0_). The inhibition constant K_I_ was determined using the weighted linearized dependence of the derived K_Mapp_ values on the inhibitor concentration [I]_0_ according to Equation (1). The weights for K_Mapp_ were set to the reciprocal squares of their estimated standard deviations.

(1) $$K_{\mathrm{Mapp}}=K_{M}\left(1+\frac{{\left[I\right]}_{o}}{K_{I}}\right)$$

### 4.4. Bacterial Strains Used for Studying Unithiol Inhibition Activity against the Metallo-β-Lactamases NDM-1 and VIM-2

Unithiol inhibition activity against the MBL NDM-1 was detected using *Klebsiella pneumoniae* strains 409 and 410 carrying the plasmid-localized *bla*_NDM-1_ gene. *Pseudomonas aeruginosa* clinical strains B-730P/17 and B-2099/18 carrying the *bla*_VIM-2_ gene were used to evaluate the inhibitory effect against MBL VIM-2. Phenotypic and genotypic characterization of the strains (Table 2) was completed as described previously [49]. 

Carbapenem compounds, meropenem, imipenem, doripenem, and ertapenem (Sigma, St. Louis, MO, USA) were selected for *K. pneumoniae* and *P. aeruginosa* according to recommendations of the European Committee on Antimicrobial Susceptibility Testing (Breakpoint tables for interpretation of MICs and zone diameters, Version 11.0, https://www.eucast.org/clinical_breakpoints/, accessed on 21 November 2021).

Bacterial strains were grown on a nutrient agar “GRM No. 1” (SRCAMB, Obolensk, Russia), “Mueller–Hinton Agar”, and “Mueller–Hinton Broth” (HiMedia Lab., Mumbai, Maharashtra, India) at 37 °C for 18–20 h. 

Minimum inhibitory concentrations (MICs) of β-lactams (ampicillin, ampicillin-sulbactam, cefoperazone, cefoperazone-sulbactam, cefoxitin, ceftriaxone, ceftazidime, and aztreonam), carbapenems (imipenem, meropenem, and ertapenem), fluoroquinolones (ciprofloxacin), aminoglycosides (gentamicin and amikacin), nitrofurans (nitrofurantoin), and unithiol were determined by microdilution in 96-well plates and interpreted according to the European Committee on Antimicrobial Susceptibility Testing (EUCAST, http://www.eucast.org, accessed on 21 November 2021). *E. coli* strains ATCC 25922 and ATCC 35218 were used as reference strains for assessing the quality of the nutrient medium and antimicrobials.

Genes associated with antimicrobial resistance (*bla*_TEM_, *bla*_SHV_, *bla*_CTX-M_, *bla*_OXA-48_, *bla*_NDM_, *bla*_KPC_, *bla*_VIM_, *bla*_IMP_, class 1 integrons were detected by PCR using previously described specific primers [49].

The unithiol inhibitory activity against MBLs NDM-1 and VIM-2 was detected by the disk diffusion method and the method of serial dilutions in broth.

### 4.5. Double-Disk Diffusion Test

The bacterial inoculum (the density adjusted to that of a 0.5 McFarland standard) was prepared from the overnight agar culture of the test strain in sterile saline solution and diluted three times. The Mueller–Hinton Agar (Himedia, Mumbai, India) in Petri dishes was inoculated by 1 mL of bacterial suspension and dried for 10–15 min at room temperature. Disks with carbapenems meropenem (10 μg), imipenem (10 μg), doripenem (10 μg), ertapenem (10 μg), and cefotaxime (30 μg) (Oxoid, Cambridge, UK), as a control, were put on the bacterial lawn. The disks with 470 or 3770 µg unithiol were put in the center of the Petri dish. Disks containing 730 µg EDTA were used as a positive control of the inhibitor, and saline solution as a negative control. The distance between the centers of the discs with antibiotics and inhibitors was no more than 15 mm. The test was considered positive if the inhibition zone was expanded between the antibiotic and the inhibitor disks.

### 4.6. Broth Tube Dilution Method

Minimum inhibitory concentrations (MIC) of carbapenems were determined by microdilution in 96-well plates and interpreted according to the European Committee on Antimicrobial Susceptibility Testing (EUCAST, http://www.eucast.org, accessed on 21 November 2021). *E. coli* strains ATCC 25922 and ATCC 35218 were used as reference strains.

### 4.7. Molecular Modeling

A crystal structure of the MBL NDM-1 with the hydrolyzed imipenem in the active site (PDB ID: 5YPI) [22] was used as a source of initial coordinates of heavy atoms. We substituted hydrolyzed substrate with a unithiol molecule or meropenem. Hydrogen atoms were added to a protein macromolecule assuming positively charged side chains of Lys, Arg, and negatively charged side chains of Glu and Asp; the side chain of Cys that forms a coordination bond with a zinc cation was deprotonated. Similarly, the complex of the unithiol and MBL VIM-2 was constructed by motifs of a crystal structure (PDB ID: 6J8R) [43] replacing the MS01 inhibitor with a unithiol molecule. In both cases, (2R)-2,3-disulfanylpropane-1-sulfonate stereoisomer of unithiol was utilized.

Molecular modeling protocol was the same for all considered systems. Preliminary equilibration of the model system was performed in the NAMD program package [50]. A protein molecule together with two zinc cations and a unithiol or a meropenem in the active site were solvated in the rectangular water box so that the distance between the protein surface and the border of the water box exceeded 12 Å and was neutralized by adding sodium ions. The model systems were 31,121 atoms for VIM-2–unithiol, 28,957 atoms for NDM-1–unithiol and 29,021 atoms for NDM-1–meropenem solvated complexes. Initial energy minimization was performed for 10000 steps with the CHARMM36 force field parameters for protein atoms and zinc ions [51], the TIP3P [52] parameters for water molecules, and CGenFF [53] parameters for the unithiol and meropenem. After that, the 5 ns molecular dynamics (MD) run was carried out in the NPT ensemble with 1 fs integration time step at T = 300 K. The cutoff distances were 12 Å for both electrostatic and van der Waals interactions with switching to the smoothing function at 10 Å.

Coordinates from the last frame of the classical MD trajectory were utilized as a source for the combined quantum mechanics/molecular mechanics (QM/MM) simulations. The 5 ps molecular dynamics simulations with the QM/MM potentials were performed with the following QM/MM energy minimization. The QM subsystem was described at the Kohn–Sham DFT level with the hybrid functional PBE0 [54] and D3 empirical dispersion correction [55] with 6-31G(d,p) basis sets for all atoms and LANL2DZ for zinc. The QM subsystems varied depending on the particular enzyme. For the model systems comprising MBL NDM-1, it included two zinc cations and their ligands (side chains of His250, Cys208, His189, His120, and His122), a catalytic residue Asp124, functional groups of the side chain of Lys211, Asn220, backbone of Gly219, and a water molecule that forms hydrogen bonds in the active site of the NDM-1 and a unithiol or a meropenem molecule. In a MBL VIM-2 based model system the QM part included two zinc cations and their ligands (side chains of His114, His116, His179, His240, and Cys198), side chains of catalytic Asp118, Arg205, and a unithiol molecule. All QM/MM calculations were performed using the QM/MM interface [56] between the NAMD [48] and Terachem [57] programs within the electronic embedding scheme. All 3D models are available in the Supporting Information.

## Figures and Tables

**Table 1:** The effect of unithiol on susceptibility of *K. pneumoniae* strain 410 producing metallo-β-lactamase NDM-1 to meropenem and imipenem; and on susceptibility of *P. aeruginosa* strain 730P/17, producing metallo-β-lactamase VIM-2 to doripenem and ertapenem.

Carbapenem-Producing Bacterial Strain	Carbapenem	Unithiol, mg/mL	MIC, mg/L
*Klebsiella pneumoniae* 410 (*bla*NDM-1)	Meropenem	0	>256 *
		1.5	16 *
		3.1	16 *
	Imipenem	0	32 *
		1.5	8 *
		3.1	8 *
	Unithiol without antibiotic		6300 **
*Pseudomonas aeruginosa* B-730P/17 (*bla*VIM-2)	Doripenem	0	32 *
		1.5	8 *
		3.1	8 *
	Ertapenem	0	32 *
		1.5	8 *
		3.1	8 *
	Unithiol without antibiotic		6300 **

Note: *—MIC of antibiotic; **—MIC of unithiol.

**Table 2:** Characterization of *K. pneumoniae* and *P. aeruginosa* strains used in this study.

Bacterial Strain	Collection Year	Isolation Source	Phenotype of the Antibacterial Resistance	Antibacterial Resistance Genes
*K. pneumoniae* 409	2013	Trachea	AMP, SAM, CEP, CFS, FOX, CRO, CAZ, ATM, IPM, MEM, CIP, GEN, AMK, NIT	*bla*_NDM,_*bla*_SHV_, *bla*_TEM_, *bla*_CTX-M_
*K. pneumoniae* 410	2013	Trachea	AMP, SAM, CEP, CFS, FOX, CRO, CAZ, ATM, IPM, MEM, CIP, GEN, AMK, NIT	*bla*_NDM_ *, bla*_SHV_ *, bla*_TEM_ *, bla*_CTX-M_
*P. aeruginosa* B-730P/17	2017	Trachea	AMP, SAM, CEP, CFS, FOX, CRO, CAZ, MEM, EPM, CIP, GEN, AMK, NIT	*bla*_VIM-2_, *int1*
*P. aeruginosa* B-2099/18	2018	Trachea	AMP, SAM, CEP, CFS, FOX, CRO, CAZ, MEM, EPM, CIP, GEN, AMK, NIT	*bla*_VIM-2_, *int1*

Note: AMP, ampicillin; SAM, ampicillin—sulbactam; CEP, cefoperazone; CFS, cefoperazone—sulbactam; FOX, cefox-itin; CRO, ceftriaxone; CAZ, ceftazidime; ATM, aztreonam; IPM, imipenem; MEM, meropenem; EPM, ertapenem; CIP, ciprofloxacin; GEN, gentamicin; AMK, amikacin; NIT, nitrofurantoin; blaSHV, blaTEM, blaCTX-M, blaNDM, blaVIM-2—β-lactamase genes; int1—class 1 integrase gene.

## Data Availability

Data is described in the main text and the Supplementary Materials.

## Supplementary Materials

The following supporting information can be downloaded at: https://www.mdpi.com/article/10.3390/ijms23031834/s1.

## Abbreviations

DBO: diazabicyclooctans; MBL, metallo-β-lactamase; BAL, British Anti-Lewisite (dimercaprol); EDTA, ethylenediaminetetraacetic acid; MIC, minimum inhibitory concentration; NMR, nuclear magnetic resonance; QM/MM, quantum mechanics/molecular mechanics; ES, enzyme substrate complex; EI, enzyme inhibitor complex.
