# Supplementary material for: Drug Repurposing of the Unithiol: Inhibition of Metallo-β-Lactamases for the Treatment of Carbapenem-Resistant Gram-Negative Bacterial Infections
Source: Int J Mol Sci. 2022 Feb 6;23(3):1834. doi: 10.3390/ijms23031834 (PMC8837113; doi:10.3390/ijms23031834)
Supplement: Supplementary file 1 [file ijms-23-01834-s001.zip › ijms-1520122-supplementary.pdf]

Supporting Information for

Drug Repurposing of the Unithiol: Inhibition of Metallo-β-Lactamases for the Treatment of Carbapenem-Resistant Gram-Negative Bacterial Infections

Vitaly G. Grigorenko [1,*], Maria G. Khrenova [1,2,*], Irina P. Andreeva [1], Maya Yu. Rubtsova [1], Anastasia I. Lev [3,†], Tatiana S. Novikova [3], Elena V. Detusheva [3], Nadezhda K. Fursova [3], Ivan A. Dyatlov [3] and Alexey M. Egorov [1]

[1] Department of Chemistry, Lomonosov Moscow State University, 119991 Moscow, Russia; Imtek1@mail.ru (I.P.A.); mrubtsova@gmail.com (M.Y.R.); alex.m.egorov@gmail.com (A.M.E.)

[2] Bach Institute of Biochemistry, Federal Research Centre "Fundamentals of Biotechnology" of the Russian Academy of Sciences, 119071 Moscow, Russia

[3] State Research Center for Applied Microbiology & Biotechnology, 142279 Obolensk, Russia; anastasia.lev@weizmann.ac.il (A.I.L.); pozitifka.15@yandex.ru (T.S.N.); klub@bk.ru (E.V.D.); n-fursova@yandex.ru (N.K.F.); dyatlov@obolensk.org (I.A.D.)

* Correspondence: vitaly.grigorenko@gmail.com (V.G.G.); mkhrenova@lcc.chem.msu.ru (M.G.K.)

† Current address: Feinberg Graduate School, Weizmann Institute of Science, Rehovot 76100, Israel.

Equilibrium geometry configurations obtained in this study are available at ZENODO at https://doi.org/10.5281/zenodo.3941468.

Table S1. Structures of potential inhibitors, initial hydrolysis rates of CENTA by metallo-β-lactamase NDM-1 in the presence of selected compounds, and relative activities. The concentration of the studied compounds in the reaction mixture was 100 µM.

**Compound**	**Structure**	**Vo, µM/s**	**Relative activity, %**
**NDM-1,** β-lactamase, control	-	0.86±0.11	100
**Unithiol** (Dithiolpropanesulfonate sodium salt)	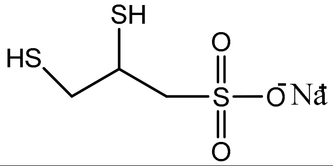	0.23±0.03	27±3
**EDTA** (Ethylenediaminetetraacetic acid)	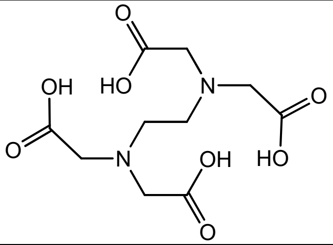	0.56±0.07	65±9
**L-Captopril** ((*2S*)-1-[(*2S*)-2-methyl-3-sulfanylpropanoyl]-pyrrolidine-2-carboxylic acid)	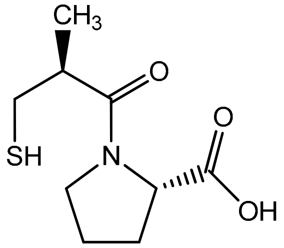	0.68±0.09	79±10
**ACC** (N-ацетил-L-цистеин, (*R*)-2-acetamido-3-sulfanylpropanoic acid)	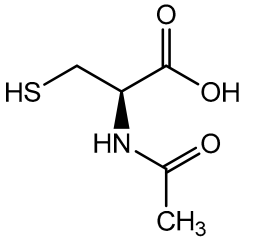	0.78±0.1	91±12
**Pentetate calcium trisodium** (Diethylenetriaminepentaacetic Acid Calcium Trisodium Salt)	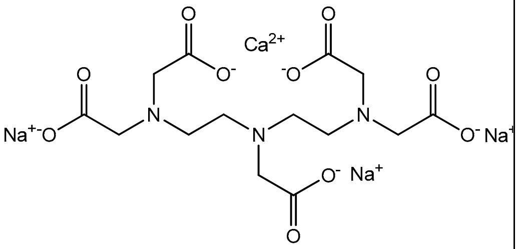	0.77±0.09	90±11
**Pamidronic acid** (3-amino-1-hydroxy-1 phosphono propyl)-phosphonic acid	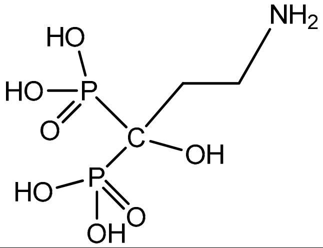	0.79±0.1	92±12
**Bisphosphonates (compounds 1-7)**	See Table 2s		

Table S2. Structures of selected bisphosphonates*, initial hydrolysis rates of CENTA by metallo-β-lactamase NDM-1 and relative activities in the presence of these compounds. The concentration of the studied compounds in the reaction mixture was 100 μM.

*Compound, N	Structure	Vo, μM/s	Relative activity, %
1	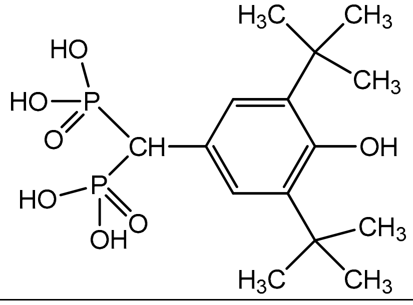	0.91±0.12	105±13
2	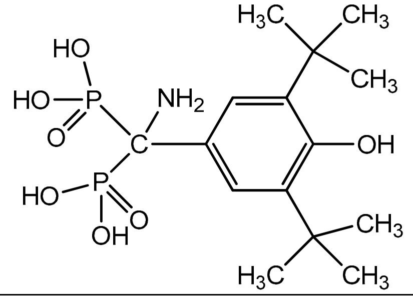	0.82±0.11	95±12
3	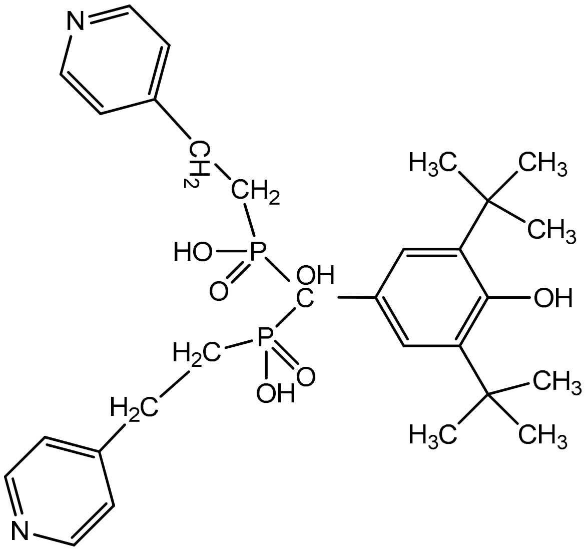	0.83±0.10	96±13
4	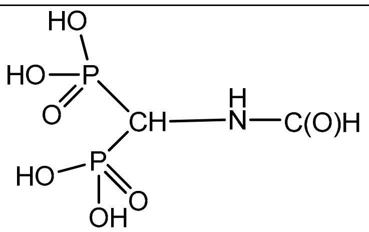	0.81±0.10	94±12
5	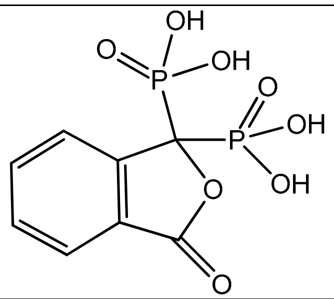	0.68±0.08	79±10

**6**	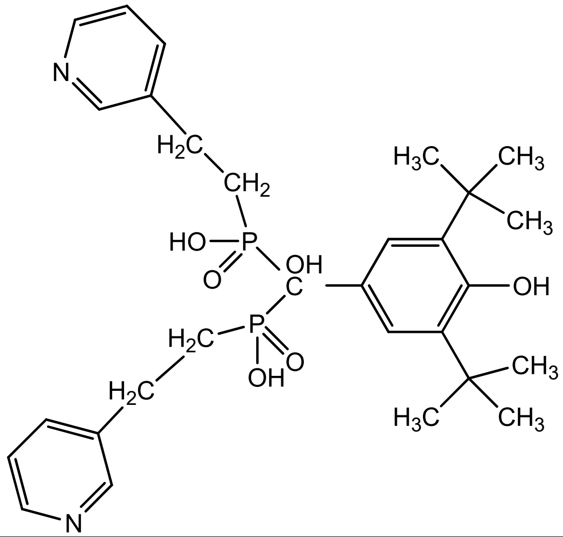	0.77±0.08	89±11
**7**	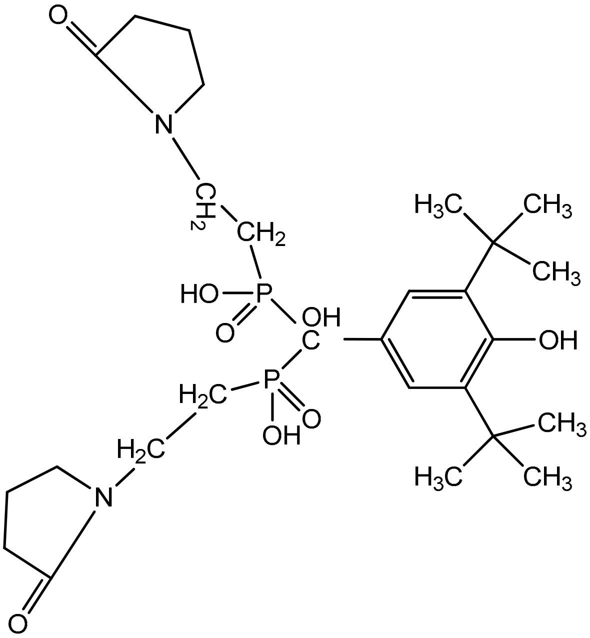	0.81±0.10	94±12

*** -** Bisphosphonates were kindly provided by Dr. Andrey A. Prishchenko, Department of Chemistry, M.V. Lomonosov Moscow State University, Moscow, 119991, Russia

Experimental conditions were as follows:

The activity of metallo-β-lactamase NDM-1 was determined for the substrate CENTA using UV-1602 spectrophotometer (Shimadzu, Japan) at 25°C in 50 mM sodium-phosphate buffer solution (pH 7.0). Product formation was detected at the wavelength of 405 nm ($\Delta\varepsilon 405 = 6400 M^{-1} cm^{-1}$). To screen the compounds against their inhibition potency, the initial rates of the enzymatic reaction (V_0, µM/s) were calculated using the initial linear part of the kinetic curve of accumulation of hydrolysis product. Concentrations of the enzyme, CENTA substrate and the inhibitors were 0.015 µM, 50 µM and 100 µM, respectively. All measurements were made in triplicate.

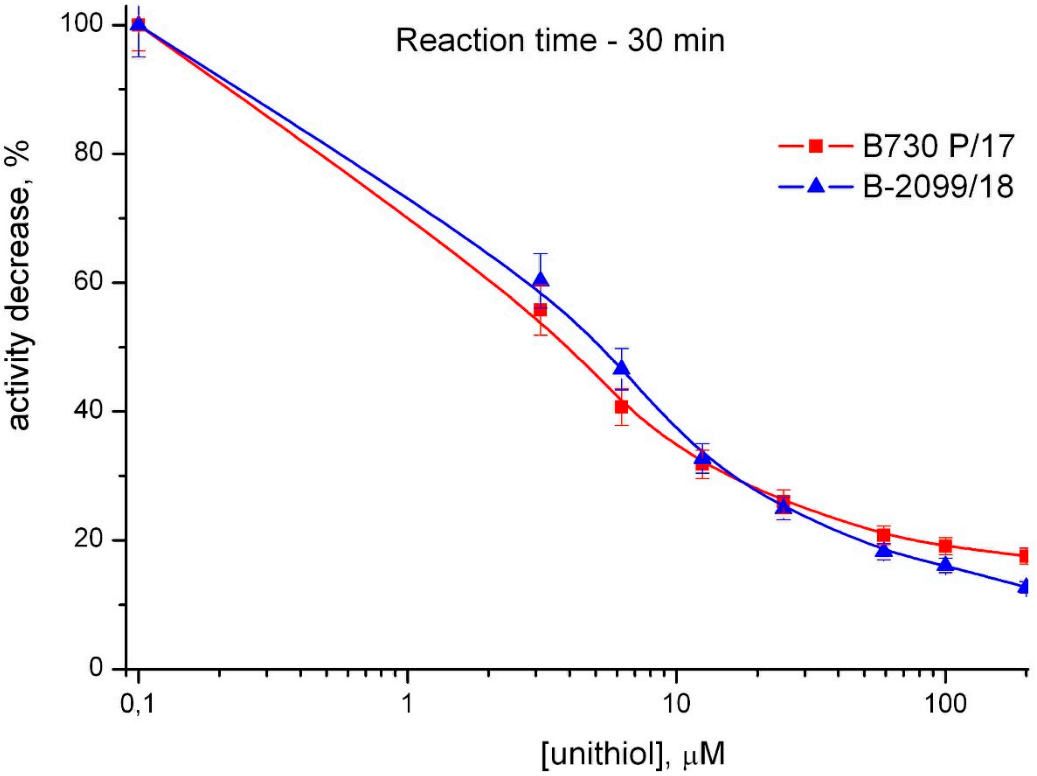

Figure S1. Dependence of the rate of CENTA hydrolysis by metallo-β-lactamase VIM-2 produced by *P. aeruginosa* strains B-730P/17 and B-2099/18 on the concentration of unithiol.

Experimental conditions were as follows:

The enzymatic activity of metallo-β-lactamase VIM-2 against CENTA was measured in 50 mM sodium phosphate buffer, pH 7.0 at 25°C, in 96-well microtiter plate format, using a Thermo Scientific Multiscan FC spectrophotometer. CENTA was prepared as stock solution of 4 mM in 50 mM sodium phosphate buffer, pH 7.0. The total volume of the enzyme assay was 200 μl. Concentration of unithiol was 0, 3.125, 6.25, 12.5, 25, 50, 100, 200 μM in the well. The concentration of CENTA was 100 μM. Each well contained 5 μl of periplasmic fraction, isolated from *P. aeruginosa* strains B-730P/17 and B-2099/18. The hydrolysis of CENTA was monitored by periodic recording of the absorbance (every 3 min during 30 min) at 405 nm ($\Delta\varepsilon 405 = 6400 M^{-1} cm^{-1}$) [C. Bebrone, C. Moali, F. Mahy, S. Rival, J.D. Docquier, G.M. Rossolini, J. Fastrez, R.F. Pratt, J.M. Frère, M. Galleni, CENTA as a Chromogenic Substrate for Studying β-Lactamases, Antimicrob. Agents Chemother. 45 (6) (2001) 1868-1871].
